# Activation of p38 MAPK participates in the sulbactam-induced cerebral ischemic tolerance mediated by glial glutamate transporter-1 upregulation in rats

**DOI:** 10.1038/s41598-020-77583-0

**Published:** 2020-11-26

**Authors:** Xiao-Hui Xian, Jun-Xia Gao, Jie Qi, Shu-Juan Fan, Min Zhang, Wen-Bin Li

**Affiliations:** 1 Department of Pathophysiology, Hebei Medical University, 361 Zhongshan East Road, Shijiazhuang, 050017 People’s Republic of China; 2 Neuroscience Research Center of Hebei Medical University, Shijiazhuang, People’s Republic of China

**Keywords:** Stroke, Transporters in the nervous system

## Abstract

Our previous studies have shown that sulbactam can play a neuroprotection role in hippocampal neurons by upregulating the expression and function of glial glutamate transporter-1 (GLT-1) during ischemic insult. Here, using rat global cerebral ischemia model, we studied in vivo the role of p38 mitogen-activated protein kinases (MAPK) in the sulbactam-induced GLT-1 upregulation and neuroprotection against ischemia. The hippocampal CA1 field was selected as observing target. The expressions of phosphorylated-p38 MAPK and GLT-1 were assayed with western blot analysis and immunohistochemistry. The condition of delayed neuronal death (DND) was assayed with neuropathological evaluation under thionin staining. It was shown that administration of sulbactam protected CA1 hippocampal neurons against ischemic insult accompanied with significantly upregulation in the expressions of phosphorylated-p38 MAPK and GLT-1. The time course analysis showed that sulbactam activated p38 MAPK before the GLT-1 upregulation in either normal or global cerebral ischemic rats. Furthermore, inhibiting p38 MAPK activation by SB203580 blocked the GLT-1 upregulation and neuroprotection induced by sulbactam. The above results suggested that p38 MAPK, at least partly, participated in the sulbactam-induced brain tolerance to ischemia mediated by GLT-1 upregulation in rats.

## Introduction

A variety of studies have shown that cerebral ischemia and hypoxia may cause a great deal of glutamate release to the synaptic gap during ischemic stimulation and the glutamate excitotoxicity is a considerable mechanism involved in ischemic neuronal damage^1,2^. The homeostasis of the glutamate concentration in the synaptic gap is mainly dependent on GLT-1^3^. Our previous studies showed that upregulation of GLT-1 by cerebral ischemic pretreatment could alleviate ischemic injury on CA1 hippocampal pyramidal neurons^4,5^. Therefore, modulating expression and function of GLT-1 might provide a new way to prevent and therapy the cerebral ischemic disease.

Rothstein et al. have reported the role of ceftriaxone in promoting GLT-1 expression and enhancing its ability to clear the glutamate from synaptic gap, which was of great benefit to alleviate disorders associated with over-accumulation of glutamate such as ischemic neuronal damage^6–8^. However, in view of the side effects such as bacterial resistance and dysbacteriosis caused by extensive use of antibiotics, it is greatly limited using ceftriaxone to prevent and therapy the cerebral ischemic damage. Sulbactam is an atypical β-lactam antibacterial and structurally similar to ceftriaxone, but has minor antibacterial activity. It might be potentially valuable for clinical application in prevent and therapy brain ischemic disease if sulbactam has anti-ischemic effect as well. Recent studies of our team showed that pretreatment with sulbactam could improve GLT-1 protein expression as well in CA1 hippocampus of rats, and enhance the viability of neurons against the subsequent global cerebral ischemia^9^. However, the mechanism about the upregulation of GLT-1 during the process of sulbactam pretreatment is not yet fully known.

The p38 MAPK involves in many physiological and pathophysiological processes, such as cell death and survival^10–12^. Recently, we found in vitro study that activation of p38 MAPK mediated the sulbactam-induced GLT-1 upregulation and neuronal protection against ischemic insult^13^. Hereby, we established the present study in vivo to further confirm the role of p38 MAPK activation in the sulbactam-induced GLT-1 upregulation and neuronal protection against ischemic insult using a rats’ global cerebral ischemia model.

## Material and methods

### Animal and groupings

Adult rats (Wistar, male, 280–320 g) were ordered from the Experimental Animal Center of Hebei Medical University. All the rats were fed in a room with an alternation of 12 h light and dark, took food and water freely. The animal care and experimental protocols were carried out according to the ‘ARRIVE’ guidelines and accepted by the Committee of Ethics on Animal Experiments of Hebei Medical University. All efforts were conducted to reduce the suffering and quantity of animals.

### Part 1: the impact of p38 MAPK in GLT-1 protein upregulation in the hippocampal CA1 region induced by sulbactam

For this purpose, it was conducted to compare the time courses of the upregulations between phosphorylated-p38 MAPK and GLT-1 with the pretreatment of sulbactam and to explore the effect of inhibiting p38 MAPK with SB203580, a specific p38 MAPK inhibitor, on the upregulation of GLT-1 induced by sulbactam pretreatment. Seventy-five rats were separated into 7 groups randomly: ① sham, ② cerebral ischemia, ③ sulbactam + sham, ④ sulbactam + cerebral ischemia, ⑤ SB203580 + sulbactam + sham, ⑥ SB203580 + sulbactam + ischemia and ⑦ SB203580 + sham groups.

**Figure 1:**
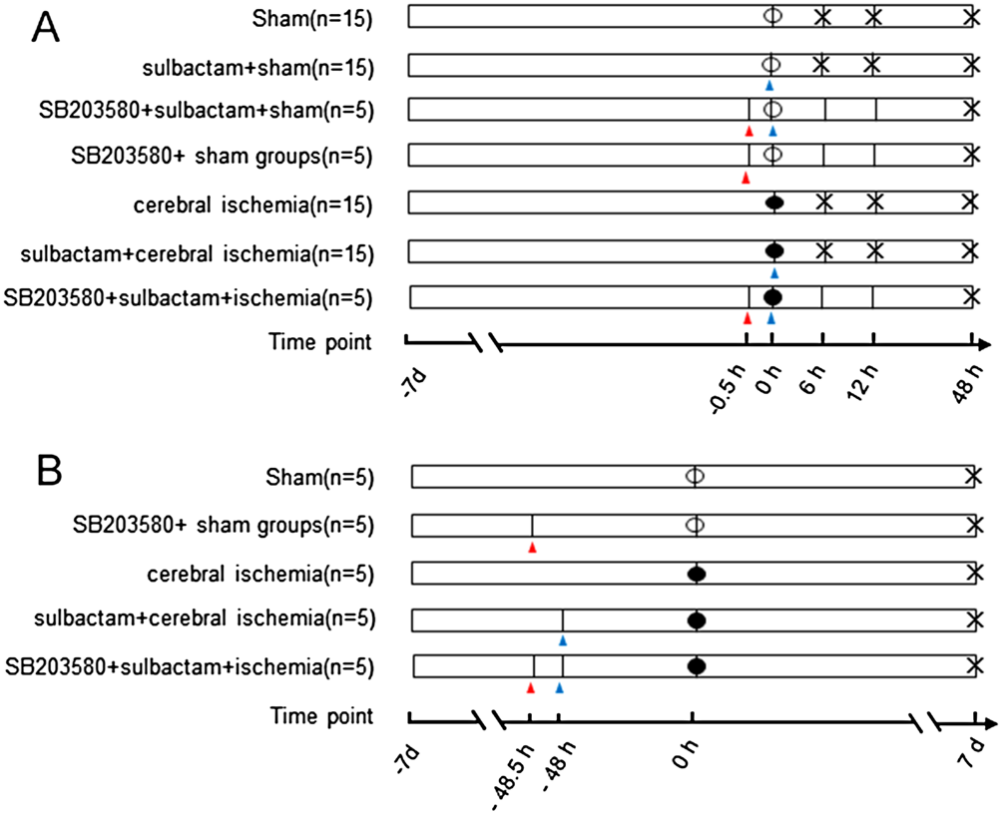
The flowchart shows the experimental protocol of Part I (**A**) and part II (**B**). The 0 h time point indicates the initiation of global brain ischemia for 8 min (●) or it’s sham operation (○). The negative time point left means before the 0 h time point and the time point right means later. The blue arrowheads indicate the time points for the lateral cerebroventricular injection of sulbactam or its vehicle (*NS* normal saline). The red arrowheads indicate the time points for the lateral cerebroventricular injection of SB203580. The symbols “ × ” in each group indicate the sampling times. The numbers in each group indicate the total number of animals.

The protocol of treatment for each group is shown in Fig. 1 A. In groups of ③ and ④, sulbactam (360 nmol dissolved in 10 μl normal saline) was administrated to right cerebral ventricle via beforehand implanted cannula first, and then global cerebral ischemia for 8 min or its sham operations were performed immediately in corresponding group, while in group of ① and ②, as a solvent control for sulbactam, normal saline in the same volume was administrated via the same root before the global cerebral ischemia or its sham operations. The rats in groups of ①–④ were sacrificed at 6 h, 12 h and 48 h (n = 5 for each time point) after the pretreatment of sulbactam or normal saline to evaluate and compare the time courses between phosphorylated-p38 MAPK and GLT-1 expressions by western blot and immunohistochemistry analysis.

In groups of ⑤–⑦(n = 5 in each group), SB203580(5 nmol, 10 μl), a specific inhibitor of p38 MAPK, was first administrated via beforehand implanted cannula 30 min before sulbactam administration. Then the rats were treated in the same protocols with the group of ③, ④ and sham, respectively, and sacrificed by decapitation under isoflurane anesthesia at 48 h after the administration of sulbactam when the upregulation of GLT-1 is relative obvious according to our preliminary experiment.

### Part 2: the impact of p38 MAPK inhibition on sulbactam-induced protection of CA1 hippocampal pyramidal neurons

Twenty-five rats were separated into the following 5 groups randomly (n = 5 in each group): ①Sham, ②cerebral ischemia, ③sulbactam + ischemia, ④SB203580 + sulbactam + ischemia, and ⑤SB203580 + sham groups. The treatments in groups of ①–④ were the same with the corresponding group in part 1, except the administration of sulbactam or normal saline was conducted 2 days before the global cerebral ischemia or its sham operations. In group ⑤ SB203580 was administrated via beforehand implanted cannula 2 days before the sham operations for global cerebral ischemia. Rats were sacrificed by decapitation under isoflurane anesthesia on 7 day after the global cerebral ischemia or its sham operations to evaluate the condition of DND by neuropathological evaluation (Fig. 1B).

### Cannula implantation

Under anesthesia by isoflurane, rats were localized in a stereotaxic apparatus (68025, Reward, China). A hole was drilled in the specified position of skull (1.5 mm right side to the midline, 0.8 mm backward to the bregma) using a dental drill with high speed (XL-30 W, OSADA, USA) according to the rat stereotaxic atlas of brain. A cannula with an inner core (Reward, China) was vertically inserted into the right lateral cerebral ventricle (3.7–4.0 mm depth to the skull surface) after incised the dura carefully, and fixed on the skull using dental cements. The rats were kept in a single cage at least 5 days after the implanting for recovery.

### Global cerebral ischemia

Four-vessel occlusion method was used to establish the rat model of global cerebral ischemia according to the reports before^14^. To the benefit of rat’s recovery from surgical stimulation, the ischemic model was established by two steps. First, the bilateral vertebral arteries of the rats were electrocoagulated under isoflurane anesthesia. A dorsal surgical incision of 1.5 cm overlying the atlas directly was performed. After exposed the atlas carefully, a preheated electrocautery needle was used to electro-coagulate bilateral vertebral arteries permanently via the alar foramen. After two days recovery, the rats’ bilateral common carotid arteries were exposed under anesthesia of isoflurane and clamped by clips for 8 min to occlude the blood flow in them to produce global cerebral ischemia, which usually result in DND of pyramidal neurons of hippocampus CA1 region^15^. Because it has been reported that systemic anesthesia can protect cerebral neurons against ischemic insult^16^, to exclude the effect of the anesthetic on the neuronal survival after ischemic insult and to evaluate the occurrence of global cerebral ischemia, the clamping of the bilateral common carotid arteries was performed when the rats awaked from the anesthesia of isoflurane. To prevent pain during the process free of the isoflurane anesthesia, the wound was continuously treated with procaine. Changes on consciousness, pupils, and light reflex were examined to evaluate the condition of the rats. During the global cerebral ischemia, the consciousness of the rat completely lost, thus the neurological behavioral deficits could not be observed. Only rats with complete loss of consciousness and light reflex with pupils dilated completely after the clamping were used for further treatments. The success rate of the model in this study is about 70–80%. After the occlusion, the clips were removed to resume the blood flow of the bilateral common carotid arteries. The consciousness and other neurological functions of the rats completely recovered after the blood flow was restored. The wound was sutured after the treatments. A temperature controllable electric-blanket was used to keep rats’ body temperature at around 37 °C throughout the process. A temperature probe is placed inside the animal's anus to detect the body temperature and the device automatically regulated the temperature of the blanket to maintain the body temperature around 37 °C.

The sham operations for the global cerebral ischemia included all the treatments mentioned above except occluding the bilateral vertebral arteries and common carotid arteries.

### Intracerebroventricular injection

Instead of the inner core, a needle was inserted in the guide cannula. At the determined time point, SB203580 (10 μl, 5 nmol) or sulbactam (10 μl, 360 nmol) or NS (10 μl) was respectively administrated using a micro syringe connecting to the needle over a 20-min period. To avoid overflowing, additional 10 min was set to keep the needle in the cannula after the administration.

### Western blotting

Rats were executed at the determined time mentioned above. The CA1 region of the right hippocampus was isolated and homogenized in 10 volume lysis buffer^17,18^. After centrifugation, the protein concentration in the supernatant was measured with Lowry method. Before loading to the gel, samples were mixture with loading buffer and heated at 95–100 °C for 5 min to denaturate the protein. Sample with 60 μg protein was loaded in each lane on a 10% SDS–polyacrylamide gel for electrophorse and then transferred to a PVDF membrane. After blocked the nonspecific antigen binding in TBST containing 5% skimmed milk powder for 60 min, membranes were incubated with primary antibodies at 4 °C for 12 h and secondary antibodies at 37 °C for 1 h subsequently (the details about the antibodies are followed in the supplementary files). For GLT-1, the membranes were further incubated with HRP-conjugated streptavidin at 37 °C for 1 h. The blots were detected by Amersham Imager 600 (GE, USA) and integrated optical density (IOD) was detected by AlphaEase FC gel image analysis software. Relative changes in expressions of phosphorylated-p38 MAPK protein and GLT-1 protein were represented by the IOD ratio aimed bands to that of β-actin.

### Immunohistochemistry assay

Rats were sacrificed at the determined time mentioned above. The left part of the brain was removed and paraffin sections of 5-μm thickness were prepared firstly. After xylene dewaxing and gradient alchohol hydration, sections were incubated with 3% H_2_O_2_ at room temperature for 15 min to remove the influence of endogenous peroxide. Sequentially, the sections were incubated with primary antibodies for 24 h at 4 °C after 1 h incubation with 10% normal goat serum. Then sections were incubated with secondary antibodies at 37 °C for 50 min after washing with PBS 3 times. HRP-conjugated streptavidin was used as third antibody and DAB substrate kit was used to develop the peroxidase activity (the details about the antibodies are followed in the supplementary files). To ensure consistency of reaction conditions, the sections for comparison were subjected to the same immunohistochemistry run. For quantitative analysis of the immunostaining, 5 sections were randomly selected in each rat to measure the mean optical density of immunostaining using a image analysis software (Image-Pro Plus 6.0, Media Cybernetics, USA).

### Neuropathological evaluation

Rats were sacrificed 7 days after the global cerebral ischemia or its sham operations. Paraffin brain sections with thionin stain were prepared as usual. Using the methods established by Kitagawa et al. and Kato et al^15,19^, conditions of the pyramidal neurons in CA1 subfield of hippocampus were assessed by histological grade (HG) and neuronal density (ND). HG including the following 4 grades: ① grade 0, without neuronal death; ② grade I, only scattered neuronal death; ③ grade II, massive neuronal death; ④ grade III, practically all neurons dead. ND was determined by counting the numbers of intact pyramidal neurons within one optical field of high magnification in the CA1 hippocampus. Total of 3 fields were counted for each section and 3 sections were counted per animal. The average value of intact neuron numbers in the optical fields of the CA1 hippocampus was counted and presented with cell number per millimeter in the CA1 hippocampus for the ND value.

### Statistical analysis

Using SPSS 16.0 to do statistical analysis and all data were shown as mean ± standard deviation (SD) except HG. One-way ANOVA combined with Student–Newman–Keuls test as a multiple comparison method was used to test differences between groups. Kruskal–Wallis ANOVA combined with Dunn's test as a multiple comparison method was used to compare the differences between groups in HG. It was considered statistically significant with a p-value of less than 0.05. The power analysis indicates that the number of animals in each group meet the research needs.

## Results

### Sulbactam upregulated phosphorylated-p38 MAPK protein expression level before GLT-1 in CA1 hippocampus

**Figure 2:**
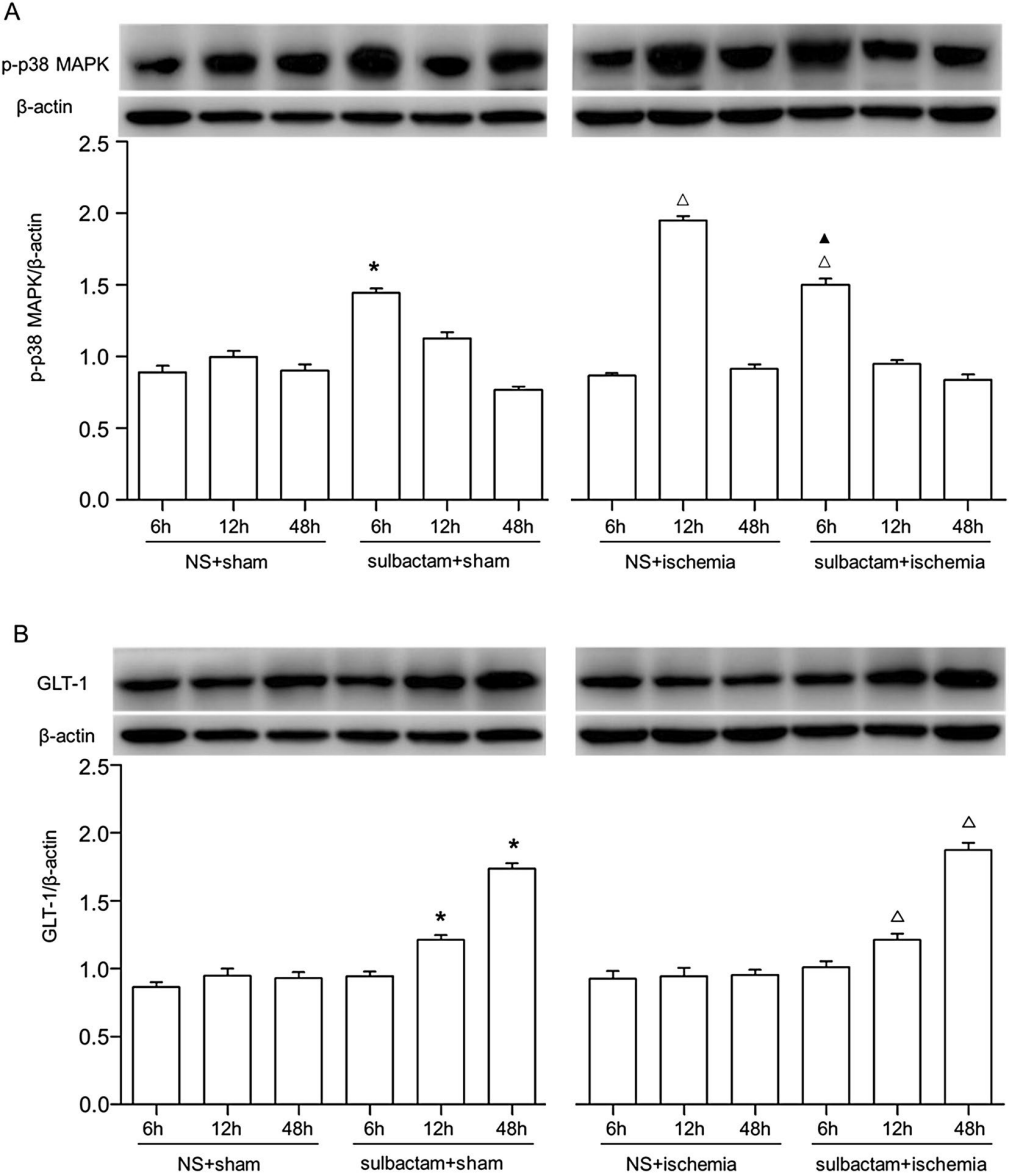
Western blot analysis shows changes in expressions of phosphorylated(p)-p38 MAPK (**A**) and GLT-1 (**B**) in the CA1 hippocampus at different time points of 6 h, 12 h and 48 h after the administration of sulbactam in sham and cerebral ischemic groups. The upper panel in (**A**,**B**) is a representative immunoblot band and the lower panel is quantitative presentation of the immunoblot bands with the ratio of integrated optical density (IOD) of the aimed protein to that of β-actin. *P < 0.05 vs NS + sham group, ^▲^*P* < 0.05 vs NS + ischemia at 12 h time point and ^∆^*P* < 0.05 vs NS + ischemia at 6 h time point. It indicated that sulbactam pretreatment upregulated the p38 MAPK activation before the GLT-1 protein upregulation in the CA1 hippocampus.

Western blot results displayed that there were basic expressions of phosphorylated-p38 MAPK protein in CA1 hippocampus in the sham rats. After sulbactam treatment, the phosphorylated-p38 MAPK expression significantly, but moderately upregulated by about 1.5-fold compared with sham level. In global cerebral ischemic rats, the phosphorylated-p38 MAPK expression obviously upregulated by more than two fold of sham level, while about 1.5-fold after the treatment of sulbactam (Fig. 2A). The GLT-1 expressions were significantly upregulated in either sham or cerebral ischemic rats after the sulbactam treatment (Fig. 2B). Cerebral ischemic insult did not induced change in the GLT-1 expression. This result is different from our previous report that the GLT-1 expression was downregulated after cerebral ischemic insult. We think that was probably due to the difference in the batch and reactivity of the animals in the present study.

In order to illustrate the relationship between the phosphorylated-p38 MAPK and GLT-1 upregulation after sulbactam pretreatment, we further compared the time sequence of their expression and found it was quite different. The expression of phosphorylated-p38 MAPK showed the most significant upregulation at 6 h after sulbactam treatment among the three observed time points and then began to fall down, while the expression of GLT-1 began to upregulate at 12 h and showed obvious upregulation at 48 h after administration of sulbactam among the three observed time points either in sham and ischemic rats. This phenomenon indicated that activation of p38 MAPK was earlier than the upregulation of GLT-1 protein expression after administration of sulbactam.

**Figure 3:**
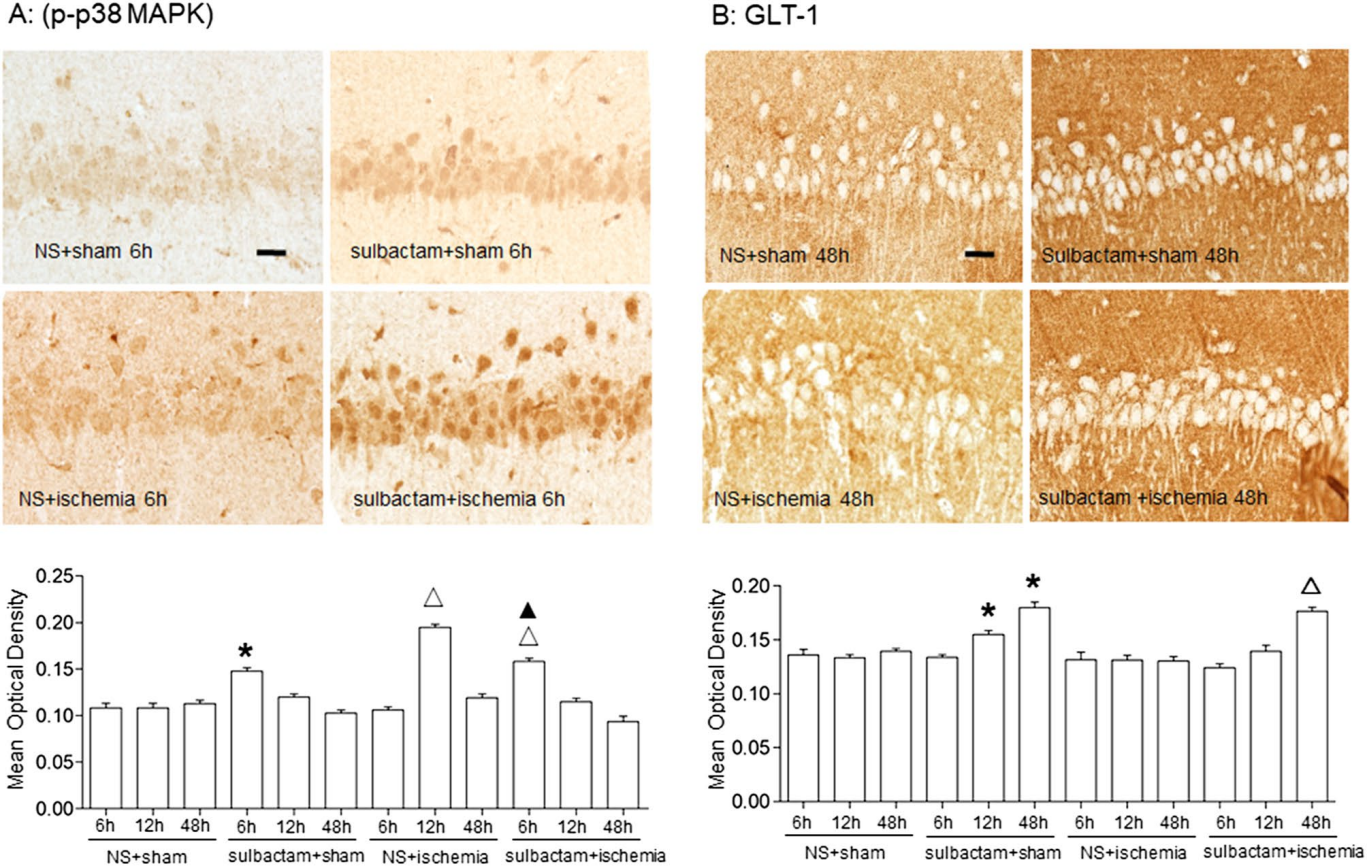
Immunohistochemistray assay shows changes in expressions of phosphorylated (p)-p38 MAPK (**A**) and GLT-1 (**B**) in CA1 region of hippocampus after administration of sulbactam. The upper is representative microphotographs of the immunostaining for p-p38 MAPK and GLT-1 after the administration of sulbactam at each time points in each group. Scale bar is 20 μm in NS + sham group and is applicable for all other microphotographs. The lower bar graph is quantitative analysis of the immunostaining with mean optical density at each time points in each group, respectively. *P < 0.05 vs NS + sham group for all time points, ^▲^*P* < 0.05 vs NS + ischemia at 12 h subgroup and ^∆^*P* < 0.05 vs NS + ischemia for all time points. It indicated that sulbactam pretreatment upregulated the p38 MAPK activation before the GLT-1 protein upregulation in the CA1 hippocampus.

Immunohistochemistry showed that there were basic expressions of phosphorylated-p38 MAPK and GLT-1 showing by light brown particles in pexiform layer, pyramidal layer and molecular layer in the CA1 hippocampus (Fig. 3). The phosphorylated-p38 MAPK immunoparticles distributed in nucleus and cytoplasm, while GLT-1 immunoparticles mainly in cytoplasm. The changes in expression levels of the proteins were similar with those indicated by western blot.

### Inhibiting the p38 MAPK activation attenuated the sulbactam-induced upregulation of GLT-1 in CA1 hippocampus

**Figure 4:**
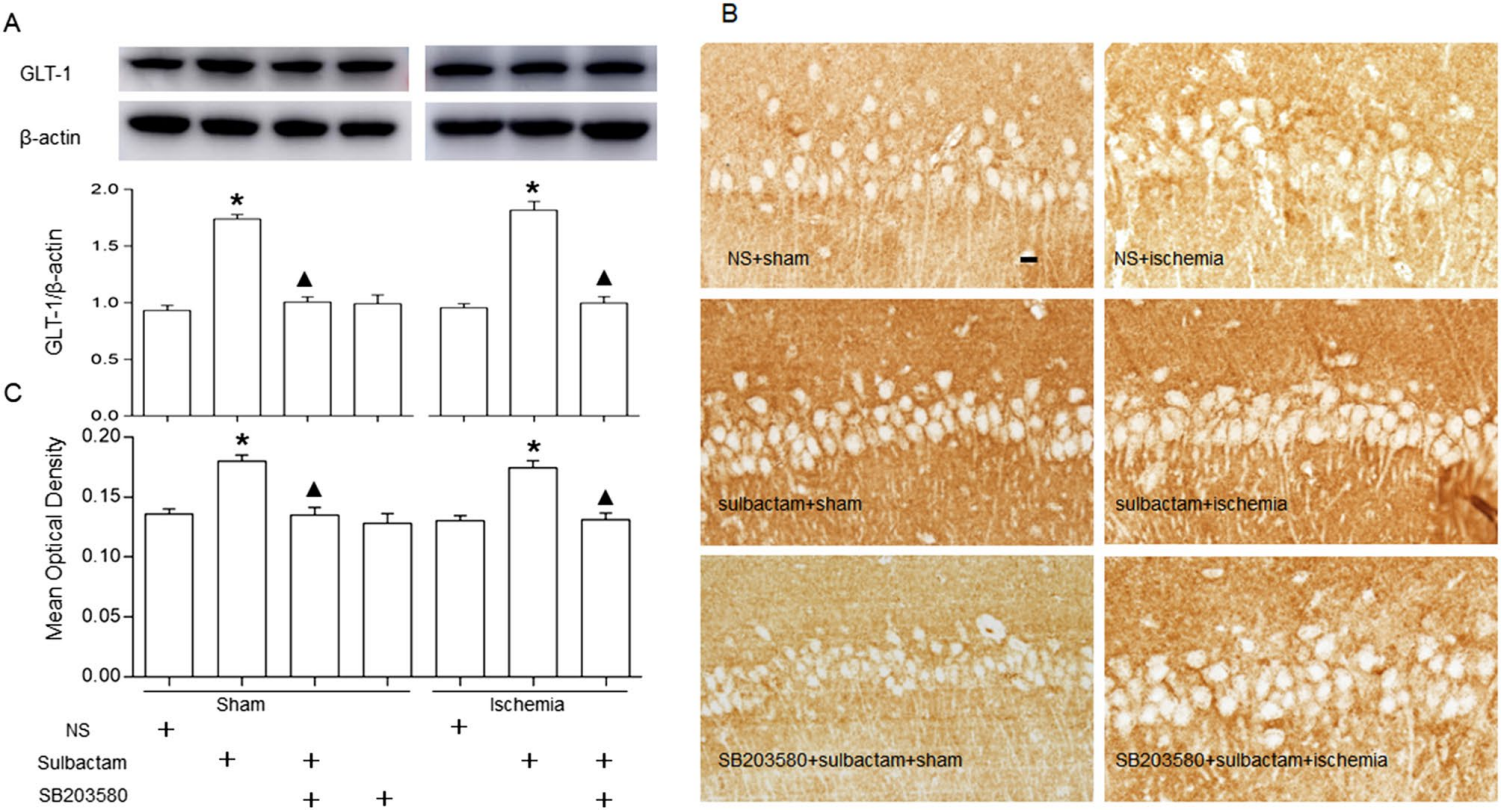
Western blot analysis and immunohistochemistray staining show effects of p38 MAPK inhibiting on upregulation of GLT-1 expression in the CA1 hippocampus at 48 h after sulbactam administration. The upper panel in (**A**) is a representative immunoblot band of GLT-1 in each group and the lower panel is quantitative presentation of the immunoblot bands with the ratio of integrated optical density (IOD) of GLT-1 to that of β-actin. The panel (**B**) is representative microphotographs of the GLT-1 immunostaining showing the GLT-1 expressions in each group. Scale bar is 20 μm in NS + sham and is applicable for all other microphotographs. The panel (**C**) is the quantitative analysis of GLT-1 immunostaining with MOD in each group. *P < 0.05 vs NS + sham group, ^▲^*P* < 0.05 vs sulbactam + sham and sulbactam + ischemia group. It indicated that the inhibition of p38 MAPK activation prevented the sulbactam-induced upregulation of GLT-1 expression in the CA1 hippocampus.

Western blot analysis (Fig. 4A) displayed that sulbactam administration significantly upregulated the GLT-1 protein expression in the CA1 hippocampus 48 h later either in sulbactam + sham or sulbactam + ischemia groups. However, pretreatment with SB203580 to block p38 MAPK phosphorylation significantly attenuated the sulbactam-induced GLT-1 upregulation either in sulbactam + sham or sulbactam + ischemia groups. Immunohistochemistry assay (Fig. 4B,C) showed similar phenomenon that compared with sham group, the increased GLT-1 immunoparticles after the administration of sulbactam were significantly attenuated by SB203580 pretreatment either in SB203580 + sulbactam + sham and SB203580 + sulbactam + ischemia group.

### Inhibiting the p38 MAPK activation prevented the sulbactam-induced neuroprotection in CA1 hippocampus

**Figure 5:**
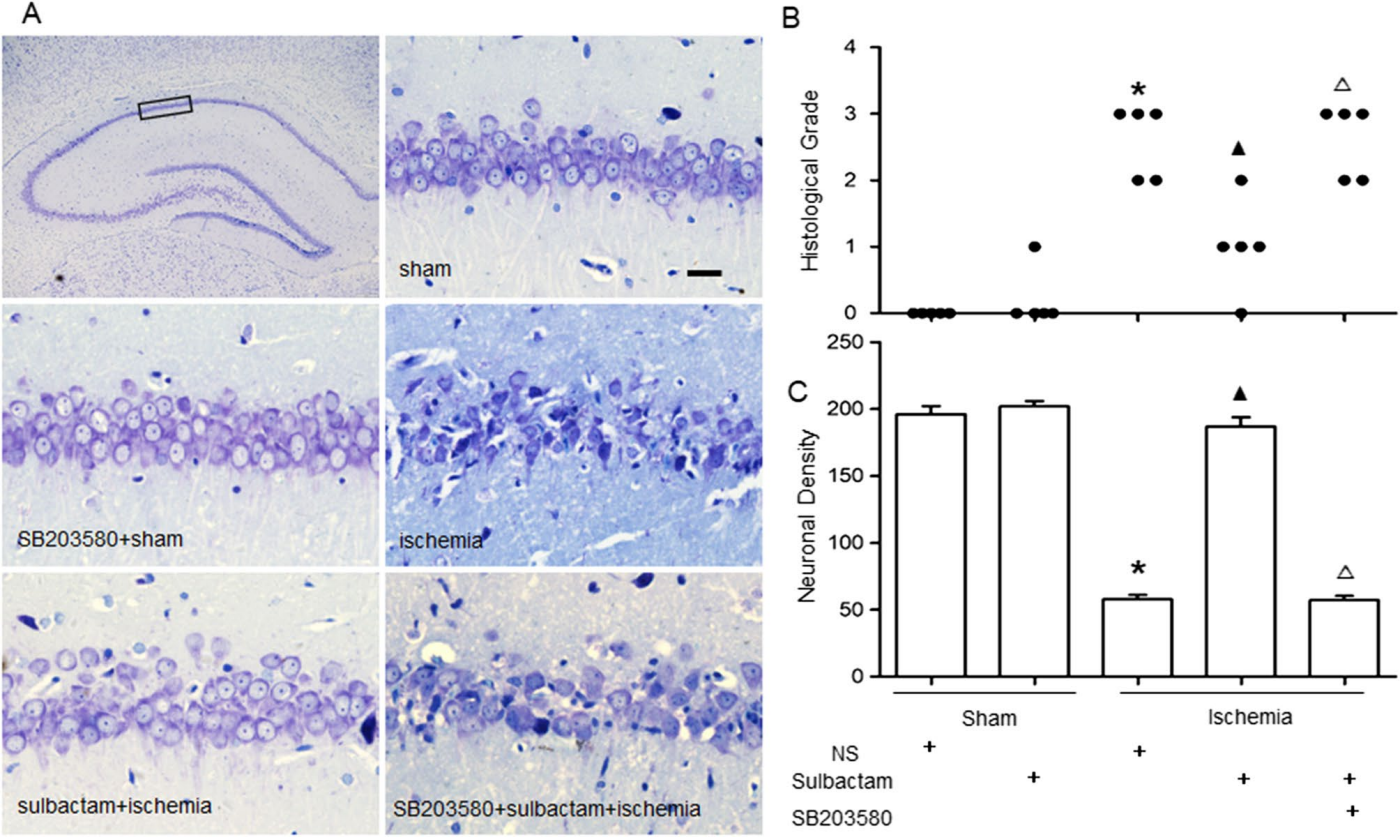
Neuropothological evaluation shows the neuronal protection of sulbactam against DND of hippocampal neurons induced by cerebral ischemic insult, and the effect of inhibiting p38 MAPK activation on the protection. The panel (**A**) is representative photomicrographs (thionin staining, × 400) of the CA1 hippocampus 7 d after the cerebral ischemic insult or sham operation. Scale bar indicates 20 μm in sham group and is applicable for all other groups. The dot and bar graphs are quantitative presentation for the histological changes in the CA1 hippocampus with HG(B) and ND(C). *P < 0.05 vs sham group, ^▲^*P* < 0.05 vs ischemia group and ^∆^*P* < 0.05 vs sulbactam + ischemia group. It indicated that sulbactam protects hippocampal neurons against ischemic insult and inhibiting the p38 MAPK activation blocked the sulbactam-induced neuroprotection in the CA1 hippocampus.

The neuropathological evaluation displayed that the pyramidal neurons arranged in order with 3–4 layers in CA1 hippocampus of sham rats (Fig. 5A). The neurons showed clear and intact contours, large number of Nissl body, and plump nucleus with clear nucleolus. The HG was grade 0 and ND was 198.6 ± 7.89. Global cerebral ischemia for 8 min resulted in large area of neuronal loss in the CA1 subfield. The remaining cells arranged disorder with irregular cell body and nuclear pyknosis. The HG increased to grade II–III and ND decreased to 51.8 ± 7.40. Pre-treatment with sulbactam in sulbactam + ischemia group notably prevented the DND induced by the global cerebral ischemia. The HG decreased to grade I–II and ND increased to about normal levels. Compared with the sulbactam + ischemia group, pretreatment with SB203580 in SB203580 + sulbactam + ischemia group blocked the neuronal protection induced by sulbactam administration, represented with significantly increase in HG to grade II–III and decrease in ND to 52.2 ± 6.61. Compared with sham group, SB203580 alone had no significant effect on the neuronal survivals in CA1 hippocampus of sham rats (SB203580 + sham group) (Fig. 5B,C).

## Discussion

Our recent study in vitro indicated that activation of p38 MAPK in cultured astrocytes mediated the sulbactam-induced GLT-1 upregulation and neuronal protection against ischemic insult^13^. The present study in vivo further confirmed the role of p38 MAPK activation in the above process using a rats’ global cerebral ischemia model, which might be particularly valuable for the establishment of the conclusion that p38 MAPK activation participates in the sulbactam-induced neuronal protection against ischemic insult via GLT-1 upregulation.

GLT-1 acts a vital role in maintaining glutamate concentration and terminating glutamatergic neurons in synaptic gap^20,21^. Studies of our team have demonstrated that upregulating GLT-1 expression and its capacity of glutamate clearance by cerebral ischemic pretreatment could alleviate the excitotoxicity induced by glutamate accompanied with neuroprotection in hippocampal neurons after cerebral ischemic attack^4,5,18^. After Rothstein’s report that ceftriaxone could selectively increase the GLT-1 levels and enhance its uptake activity^6^, the follow-up studies showed that ceftriaxone could play protective roles on diseases related with excessive glutamate. For instance, pre-treatment by ceftriaxone could increase the GLT-1 expression and relieve neuron damage in the ischemic penumbra area^22^. Post-treatment by ceftriaxone after traumatic brain injury restored GLT-1 level and reduced regional gliosis and the probability of posttraumatic seizures in rats^23^. Pretreatment with ceftriaxone in a focal cerebral ischemia model could prevent the downregulation of GLT-1 level on astrocytes and induce brain tolerance to ischemia insult^7,24^. Our recent study showed that ceftriaxone protected hippocampal neurons against global cerebral ischemia by upregulating of the GLT-1 protein level and glutamate clearance^25^. Considering the side effects of ceftriaxone such as dysbacteriosis and bacterial resistance which limit its application as an anti-ischemic medication, we further studied and showed that sulbactam, an atypical β-lactam medication, could protect hippocampal neurons against ischemic insult mediated by modulating the expression and uptake ability of GLT-1 in rats global cerebral ischemia model^9^. The present study confirmed the findings above and further reinforced the suggestion that sulbactam could play protective role for neurons against ischemic insult by upregulation of GLT-1.

On the other hand, the reverse activity of glutamate transporters might be one of the mechanisms involved in the induction of glutamate excitotoxicity in some conditions. It has been reported that knockout mice lacking GLT-1 are more vulnerable to neuronal death after a short, severe episode of ischemia than wild-type mice. While, wild-type mice expressing GLT-1 are more vulnerable to neuronal death than mice lacking GLT-1 during extended, chronic ischemia. These results indicate that GLT-1 is essential for neuroprotection after a short acute ischemia, and GLT-1 reverse activity might occur almost only when ischemia is prolonged^46^. Our previous studies have shown that cerebral ischemic preconditioning or ceftriaxone pretreatment can upregulate the GLT-1 protein expression, increase the uptake of glutamate by GLT-1, inhibit the increase of glutamate concentration, and thereby improve the survival of neurons in the CA1 region of the hippocampus in rats suffering transient global brain ischemia for 8 min which usually induces delayed neuronal death^25,47^. In the present study, the same transient global brain ischemia model was used, and the survival of hippocampal neurons was significantly improved after the pretreatment of sulbactam. This result is in consistent with our previous reports in vivo^9^ and vitro^13^. Therefore, we believe that the upregulation of GLT-1 induced by sulbactam in the present study might increase glutamate uptake of GLT-1, rather than reverse transport, and then improves neuronal survival suffering the ischemic attack by inhibiting the glutamate excitotoxicity. Further study on glutamate concentration and glutamate uptake of GLT-1 after sulbactam treatment would provide direct evidence to prove the suggestion.

Mitogen-activated protein kinases (MAPKs) belong to the serine/threonine protein kinase family and mainly consist of four members including ERK, JNK, p38 MAPK and BMK. There are 4 isoforms of p38 MAPK, named p38α, p38β (1 and 2), p38γ and p38δ. The isoforms of p38α, p38β1 and p38β2 are widely present in various tissues. The isoform of p38γ is only present in skeletal muscle cells, and p38δ is mainly present in glandular tissues. Some reports showed that both p38α and p38β isoforms of p38 MAPK were activated together in neurons or astrocytes in OGD and cerebral ischemic models although there were some differences in time course of the activation^26–28^. Therefore, it might be speculated that both p38α and p38β isoforms of p38 MAPK might be involved after the pretreatment of sulbactam in the present study.

The p38 MAPK involves in various pathophysiological processes, such as cell death and survival^10^. Previous studies have shown that activation of p38 MAPK mediates cell death. For instance, in a mouse model of focal cerebral ischemia, p38 MAPK activation was significantly increased both in the neurons and astrocytes of the ischemic region^29^. Inhibiting p38 MAPK over-activation with SB203580 or SB239063 significantly reduced DND in hippocampal CA1 region induced by cerebral ischemia^30^. However, recent studies showed that mild or moderate activation of p38 MAPK was neuroprotective in ischemia. For example, cerebral ischemic preconditioning could protect hippocampal neurons against ischemia and induced a moderate activation of p38 MAPK. Furthermore, inhibition of the moderate activation of p38 MAPK blocked the neuroprotection of cerebral ischemic preconditioning. These findings suggested that moderate or mild activation of p38 MAPK took part in the acquirement of cerebral ischemic tolerance after cerebral ischemic pretreatment^31,32^. Similar phenomena were also found in the heart, in which the cardioprotective effect induced by glycoprotein IIb/IIIa inhibitor could be blocked by inhibiting p38 MAPK phosphorylation^33^. Therefore, it is generally agreed that the activation of p38 MAPK play a dual role in the process of ischemia based on its activation level^34,35^, e.g., the over-activation of p38 MAPK resulted from fatal ischemia usually leads to neuronal damage in the brain^36^, while moderate or mild p38 MAPK activation induced by sublethal ischemia normally mediates the neuroprotective effects^37,38^. The present study showed that global cerebral ischemia obviously activated p38 MAPK by more than 2 folds of sham level accompanied with serious pyramidal DND in the CA1 hippocampus. This finding was consistent with previous studies that ischemic insult induced p38 MAPK over-activating which involved in the ischemic neuronal injury. Meanwhile, we found that pretreatment with sulbactam induced a significant but mild or moderate upregulation of p38 MAPK with magnitude of about 1.5 folds of sham levels. This phenomenon was similar with that observed in cerebral ischemic preconditioning^18^, and suggested that the moderate activation of p38 MAPK might be a mechanism involved in the neuronal protection of sulbactam.

Considering the beneficial effect of moderate or mild p38 MAPK activation in the neuroprotection mentioned above, we focused in the present study to explore the role of p38 MAPK activation in the upregulation of GLT-1 level and neuroprotection against ischemic insult induced by sulbactam. It was found that sulbactam pretreatment up-regulated both phosphorylated-p38 MAPK mildly or moderately and GLT-1 expression in the CA1 hippocampus in both normal and global cerebral ischemic rats, but the increased activation of p38 MAPK was significantly earlier than the upregulation of GLT-1 after sulbactam treatment. The results are similar with our recent study in vitro in which cultured astrocytes showed upregulation in the p38 MAPK activating before upregulating the GLT-1 protein expression after sulbactam incubation^13^, and suggested that p38 MAPK signal pathway might act as the upstream mechanism for the upregulation of GLT-1 expression after sulbactam treatment.

As for the cell types of the p38 MAPK expression in immunohistochemical figures (Fig. 3A), we believe that the elevated expression of p-p38MAPK includes neuronal and glial p-p38MAPK, because p38MAPK is a highly conserved molecule and widely expresses in the brain including neurons and glial cells, and the astrocytic p38MAPK activation mediated the upregulation of GLT-1 after the sulbactam treatment in the present study. Our previous study with double labeled immunofluorescence assay showed that the number of astrocytes expressed p-p38 MAPK significantly increased both at 6 h and 2 d after cerebral ischemic preconditioning^31^. In cultured astrocytes, sulbactam induced up-regulation of phosphorylated-p38 MAPK earlier than GLT-1 up-regulation^13^. These findings support the above suggestion. However, the present study could not provide double labeled immunofluorescence evidence to clearly indicate that the astrocytic p-p38 MAPK expression was upregulated, which indeed was a deficiency of this study and should be improved in the future study.

Many studies have shown that SB203580 could significantly inhibit the phosphorylation level of p38 MAPK, and thereby block the p38 MAPK downstream signaling cascades^39,40^. To further evaluate the role of p38 MAPK in the GLT-1 upregulation and neuroprotection against ischemic insult induced by sulbactam, we observed effects of SB203580 on expression of GLT-1 protein and conditions of DND in CA1 hippocampus after sulbactam preconditioning. It was found that after inhibiting p38 MAPK signaling pathway using SB203580, the upregulation of GLT-1 level in CA1 hippocampus normally induced by sulbactam pretreatment was attenuated both in sham or global cerebral ischemia groups. Furthermore, the sulbactam-induced neuronal protection against ischemic insult was inhibited as well by the pretreatment of SB203580. However, SB203580 alone had no effects on the GLT-1 expression and the neuron survival in the sham group. These results indicated the role of p38 MAPK activation in the process of sulbactam-induced GLT-1 upregulation and neuronal survival.

The p38 MAPK pathway is a kinase-activated cascade, and usually activated by upstream elements of MKK3/6 and SEK by phosphorylation. The p38 MAPK could be phosphorylated at several sites, e.g., T180, Y182, and Y323. Since the antibody we used in the present study is for T180 and Y182 phosphorylation sites, it could be suggested that these phosphorylation sites play a role in sulbactam-induced p38 MAPK activation, although the role of phosphorylation site of Y323 can not excluded. The activated p38 MAPK (p-p38MAPK) could rapidly transferred into the nucleus, and regulate the activation of CREB and NF-κB by MAPKAPK-2 and MSK1/2^41,42^. Several reports have showed the association of these pathways in the up-regulation of GLT-1 expression in astrocytes^43–45^. Therefore, mechanism above may be involved in the sulbactam-induced upregulation of GLT-1 in the present study, which might be evident by further studies.

Although our previous studies showed that only sulbactam pretreatment enhances neuronal resistance to ischemia, but little effect in post-treatment^9^, the pretreatment of sulbactam has certain clinical potential as well in some predictable ischemic insults such as TIA and cardiac surgery and attack which induce global brain ischemia. In these conditions, the pretreatment of sulbactam that can enhance neuronal tolerance to ischemia might be beneficial for reducing the possible neuronal damage caused by global cerebral ischemia. The current study indicated the role of p38 MAPK activation in the sulbactam-induced cerebral ischemic tolerance mediated by GLT-1 upregulation in global cerebral ischemic rats and provide further experimental evidence for the application of sulbactam in the situations above.

## Conclusion

Taken together, it is concluded that p38 MAPK, at least partly, participates in the sulbactam-induced cerebral ischemic tolerance mediated by GLT-1 upregulation in global cerebral ischemic rats.

## Supplementary information

Supplementary Information 1.

Supplementary Information 2.
