# Supplementary material for: Activation of p38 MAPK participates in the sulbactam-induced cerebral ischemic tolerance mediated by glial glutamate transporter-1 upregulation in rats
Source: Sci Rep. 2020 Nov 26;10:20601. doi: 10.1038/s41598-020-77583-0 (PMC7692545; doi:10.1038/s41598-020-77583-0)
Supplement: Supplementary file 1 — Supplementary Information 1. [file 41598_2020_77583_MOESM1_ESM.pdf]

Activation of p38 MAPK participates in the sulbactam-induced cerebral ischemic tolerance mediated by glial glutamate transporter-1 upregulation in rats

Xiao-Hui Xian[1], Jun-Xia Gao[1&], Jie Qi[1], Shu-Juan Fan[1], Min Zhang[1, 2*], Wen-Bin Li[1, 2*]
1. Department of Pathophysiology, Hebei Medical University, Shijiazhuang, P.R. China;
2. Neuroscience Research Center of Hebei Medical University, Shijiazhuang, P.R. China;

[&] Co-first author

*Correspondence: Wen-Bin Li, Department of Pathophysiology, Hebei Medical University, 361 Zhongshan East Road, Shijiazhuang 050017, China, e-mail: liwbsjz@163.com; Min Zhang, Department of Pathophysiology, Hebei Medical University, 361 Zhongshan East Road, Shijiazhuang 050017, China, e-mail: hebmuzhangmin@163.com.

Acknowledgments

This work was supported by the following foundations: National Natural Science Foundation of China (No: 81271454, 81571060) and Key Basic Research Project in Application Plan of Hebei Province of China (No:16967762D).

Antibodies for Western Blot assay

Primary antibodies：

For GLT-1: guinea-pig polyclonal antibody (1:1000, Chemicon, catalog no. AB1783).

For phosphorylated-p38 MAPK(T180/Y182): rabbit monoclonal antibody (1:500, CST, catalog no. 4631S).

For β-actin: mouse polyclonal antibody (1:1000, Santa Cruz, catalog no. SC-8432))

Secondary antibodies:

For GLT-1: biotin labeled anti-guinea-pig IgG (1:3000, Chemicon., catalog no. AP108B).

For phosphorylated-p38 MAPK: anti-rabbit IgG conjugated with IRDye700DX (1:1000, Rockland, catalog no. 611-130-122).

For β-actin: anti-mouse IgG conjugated with IRDye800CW (1:2000, Rockland, catalog no. 610-131-121).

Third antibody for GLT-1: horseradish peroxidase-conjugated streptavidin (1:2000, ZYMED, catalog no. 43-4323).

Antibodies and materials for immunohistochemistry assay

Primary antibodies

For GLT-1: guinea-pig polyclonal antibody (1:500, Chemicon, catalog no. AB1783).

For phosphorylated-p38 MAPK(T180/Y182): rabbit monoclonal antibody (1:200, CST, catalog no. 4631S).

Secondary antibodies

For GLT-1: SP0022 Bioss, China, Lot. No: AG03311135.

For phosphorylated-p38 MAPK: SP-9000, Zhongshan, China, Lot. No: WK161525.

Third antibody for GLT-1 and phosphorylated-p38 MAPK: HRP-conjugated streptavidin working solution (Lot 12127A10, Zhongshan, China).

DAB substrate kit (Lot 12196A11, Zhongshan, China)
