# Supplementary material for: Activation of p38 MAPK participates in the sulbactam-induced cerebral ischemic tolerance mediated by glial glutamate transporter-1 upregulation in rats
Source: Sci Rep. 2020 Nov 26;10:20601. doi: 10.1038/s41598-020-77583-0 (PMC7692545; doi:10.1038/s41598-020-77583-0)

Activation of p38 MAPK participates in the sulbactam-induced cerebral ischemic tolerance mediated by glial glutamate transporter-1 upregulation in rats

Xiao-Hui Xian[1], Jun-Xia Gao[1&], Jie Qi[1], Shu-Juan Fan[1], Min Zhang[1, 2*], Wen-Bin Li[1, 2*]

1. Department of Pathophysiology, Hebei Medical University, Shijiazhuang, P.R. China;

2. Neuroscience Research Center of Hebei Medical University, Shijiazhuang, P.R. China;

[&] Co-first author

*Correspondence: Wen-Bin Li, Department of Pathophysiology, Hebei Medical University, 361 Zhongshan East Road, Shijiazhuang 050017, China, e-mail: liwbsjz@163.com; Min Zhang, Department of Pathophysiology, Hebei Medical University, 361 Zhongshan East Road, Shijiazhuang 050017, China, e-mail: hebmuzhangmin@163.com.

Acknowledgments

This work was supported by the following foundations: National Natural Science Foundation of China (No: 81271454, 81571060) and Key Basic Research Project in Application Plan of Hebei Province of China (No:16967762D).

Supplementary material: the original gels of western blot

1. The western blot bands of p-p38MAPK protein in each time points of sham group and sulbactam + sham group

The lane order from left to right is:

sham 6h, sham 12h, sham 48h, sulbactam + sham 6h, sulbactam + sham 12h, sulbactam + sham 48h

blot 1

(used in Fig 2A left in the manuscript as representative blot)

p-p38

β-actin

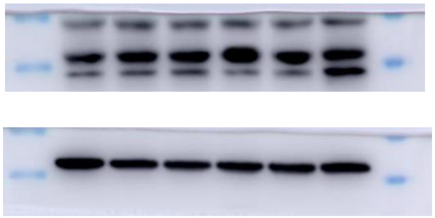

blot 2

p-p38

β-actin

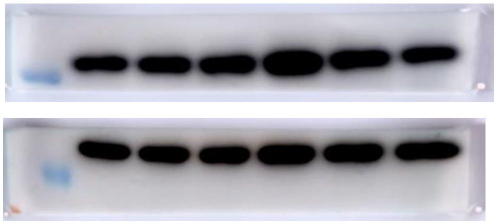

blot 3

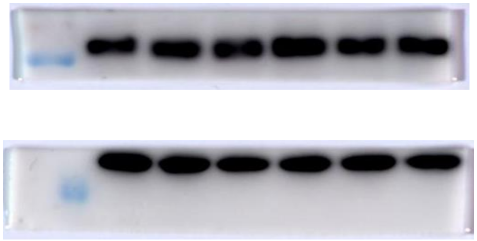

blot 4

p-p38

β-actin

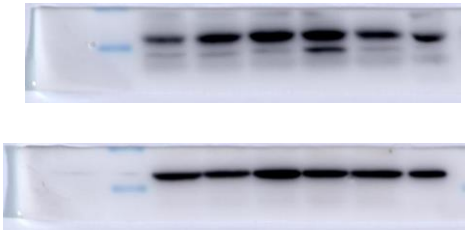

blot 5

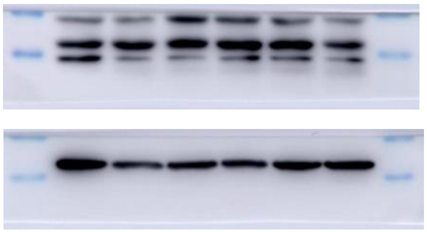

2. The western blot bands of p-p38MAPK protein in each time points of ischemia group and sulbactam + ischemia group

The lane order from left to right is:

ischemia 6h, ischemia 12h, ischemia 48h, sulbactam + ischemia 6h, sulbactam + ischemia 12h, sulbactam + ischemia 48h

blot 1

(used in Fig 2A right in the manuscript as representative blot)

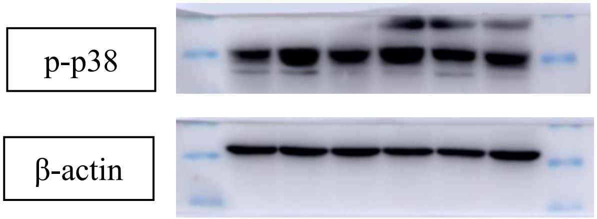

blot 2

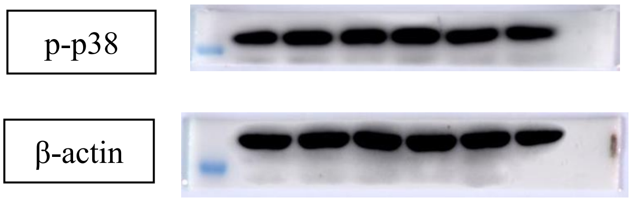

blot 3

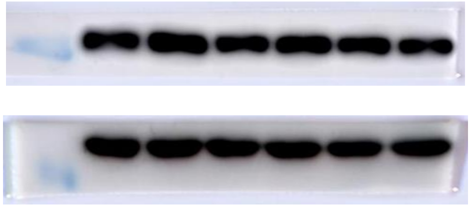

blot 4

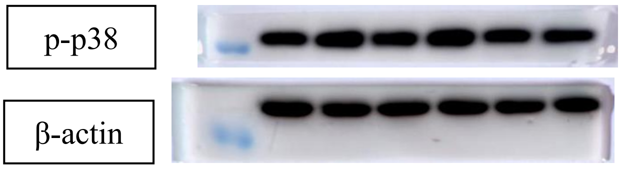

blot 5

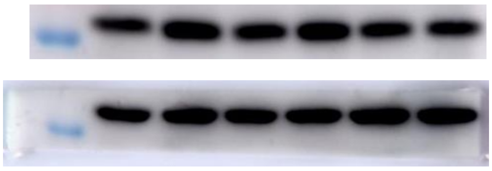

3. The western blot bands of GLT-1 protein in each time points of sham group and sulbactam + sham group

The lane order from left to right is:

sham 6h, sham 12h, sham 48h, sulbactam + sham 6h, sulbactam + sham 12h, sulbactam + sham 48h

blot 1

(used in Fig 2B left in the manuscript as representative blot)

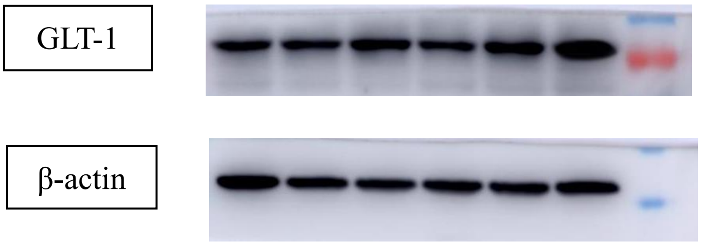

blot 2

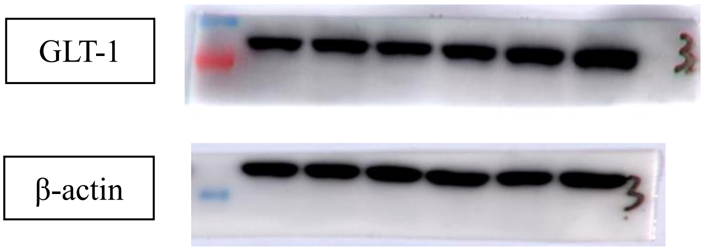

blot 3

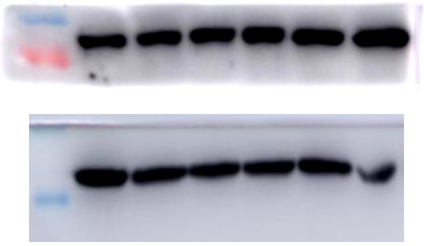

blot 4

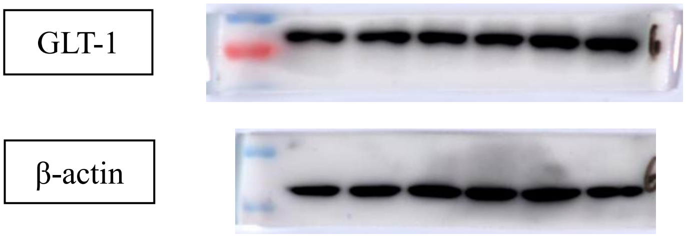

blot 5

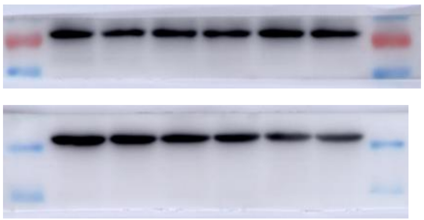

4. The western blot bands of GLT-1 protein in each time points of ischemia group and sulbactam + ischemia group

The lane order from left to right is:

ischemia 6h, ischemia 12h, ischemia 48h, sulbactam + ischemia 6h, sulbactam + ischemia 12h, sulbactam + ischemia 48h

blot 1

(used in Fig 2B right in the manuscript as representative blot)

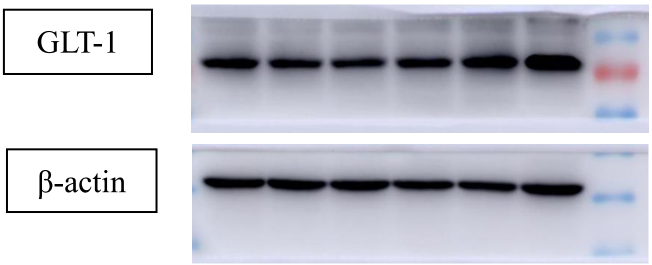

blot 2

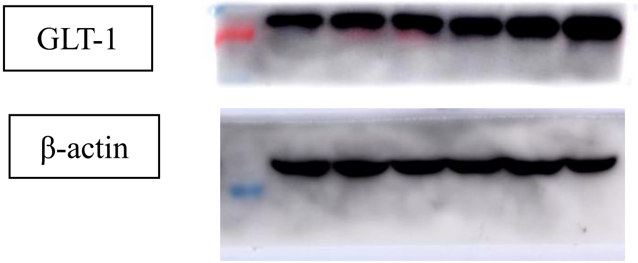

blot 3

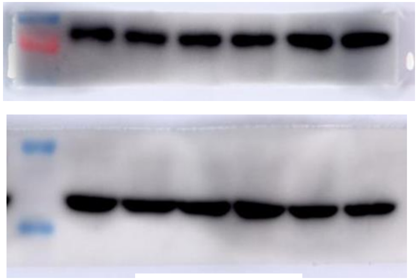

blot 4

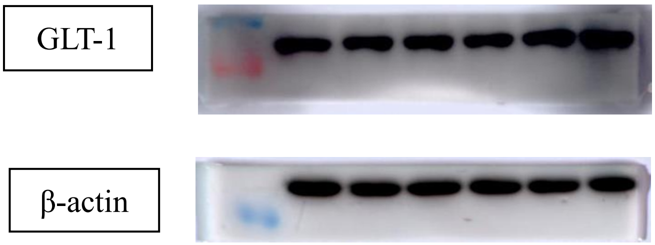

blot 5

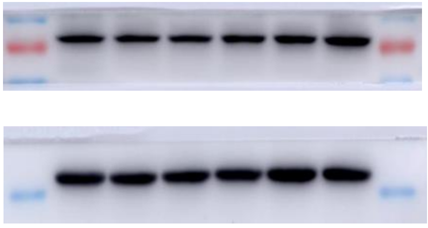

5. The western blot bands of GLT-1 protein in sulbactam pretreatment experiments with or without SB203580 present

The lane order from left to right is:

Left Panel： Sham, sulbactam + sham, SB203580 + sulbactam + sham, SB203580;

Right Panel: ischemia, sulbactam + ischemia, SB203580 + sulbactam + ischemia

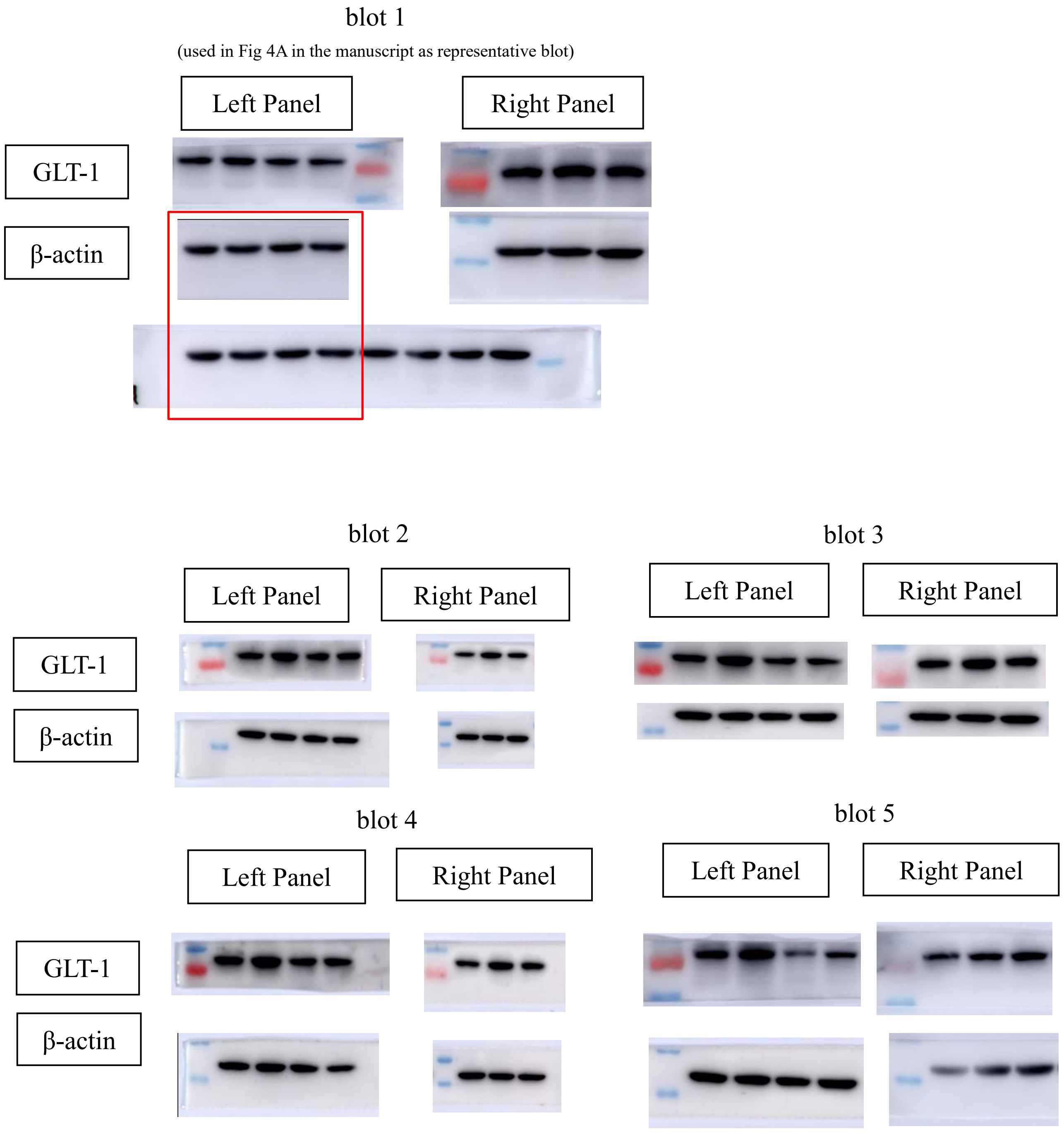

Supplement: Supplementary file 2 — Supplementary Information 2. [file 41598_2020_77583_MOESM2_ESM.pdf]
